# A systematic review of the budget impact analyses for antitumor drugs of lung cancer

**DOI:** 10.1186/s12962-020-00253-5

**Published:** 2020-12-01

**Authors:** Lu Han, Xin Zhang, Wen-Qi Fu, Cheng-Yao Sun, Xian-Ming Zhao, Liang-Ru Zhou, Guo-Xiang Liu

**Affiliations:** 1 School of Health Management/Public Health, Harbin Medical University, Harbin, 150081 China; 2 Tumor Radiotherapy Center, Harbin the First Hospital, Harbin, 150010 China

**Keywords:** Budget impact analyses, Antitumor drugs, Lung cancer

## Abstract

**Background:**

Budget impact analyses (BIAs) are used for reimbursement decisions and drug access medical insurance, as a supplement to cost-effectiveness analyses (CEAs).

**Objectives:**

We systematically reviewed BIAs for antitumor drugs of lung cancer to provide reference for high-value drug budget impact analyses and decision making.

**Methods:**

We conducted a literature search on PubMed, EMbase, The Cochrane Library, China National Knowledge Infrastructure and Wanfang Data Knowledge Service Platform from 2010 to 2019. The methodological indicators and result information of the budget impact analyses were extracted and evaluated for quality.

**Results:**

A total of 14 studies on the budget impact for antitumor drugs of lung cancer were included, and the overall quality was good. Half of studies were from developed countries. Nine of the studies were designed using the BIA cost calculation model, and two were simulated using the Markov model Monte Carlo model. From all studies, only 14.3% reported model validation. The budget impact results of the same drug in different countries were inconsistent.

**Conclusions:**

Included studies evaluating budget impact analyses for anti-tumor drugs of lung cancer showed variability in the methodological framework for BIAs. The budget impact analyses of high-value drugs need to be more stringent to ensure the accuracy of the parameters, and should provide reliable results based on real data to decision-making departments, which should carefully consider access to lung cancer drugs.

## Background

Lung cancer is the most frequent cancer and the leading cause of cancer death among males. According to the Global cancer statistics 2018, lung cancer is the most common diagnosed cancer accounting for 11.6% of the total cases, and the leading cause of cancer death accounting for 18.4% of the total cancer deaths worldwide. In 2018, there were estimated to be 2.1 million new lung cancer cases and 1.8 million deaths [1]. Meanwhile, lung cancer had the highest economic cost with €18·8 billion, 15% of overall cancer costs in the European Union [2].

The World Health Organization (WHO) divides lung cancer into non-small cell lung cancer (NSCLC) and small cell lung cancer (SCLC) based on its biology, therapy, and prognosis [3, 4]. NSCLC includes two major types that account for more than 80% of total lung cancer cases: non-squamous cell, including adenocarcinoma, large cell carcinoma, and other cell types; and squamous cell (epidermoid) carcinoma [5]. In patients with NSCLC, the most commonly found Epidermal Growth Factor Receptor(EGFR) mutations are deletions in exon 19 and a mutation in exon 21 [6]. Both mutations result in activation of the tyrosine kinase domain, and both are associated with sensitivity to the small molecule tyrosine kinase inhibitors (TKIs), such as erlotinib, gefitinib, and afatinib [7]. An estimated 2 to 7% of patients with NSCLC have anaplastic lymphoma kinase(ALK) gene rearrangements that are resistant to EGFR TKIs [8, 9].

Erlotinib and gefitinib are orally active TKIs that are very well tolerated by most patients [10, 11]. Erlotinib was approved by the FDA for the treatment of patients with locally advanced or metastatic NSCLC after progression on at least one prior chemotherapy regimen in 2014 [12]. Erlotinib and gefitinib are recommended (category 1) in the NSCLC algorithm as first-line therapy in patients with advanced, recurrent, or metastatic nonsquamous NSCLC who have known active sensitizing EGFR mutations [13–15]. Afatinib is also an oral TKI that inhibits the entire ErbB/HER family of receptors, including EGFR and HER2 [16, 17]. The FDA has approved afatinib for first-line treatment of patients with metastatic nonsquamous NSCLC who have sensitizing EGFR mutations, but its safety is slightly lower than erlotinib or gefitinib [6, 18].

Immunocheckpoint inhibitors are preferred agents recommended for subsequent treatment by NCCN. For NSCLC patients without ALK rearrangement, ROS1 rearrangement, or sensitized EGFR mutations, immune checkpoint inhibitors (nivolumab, pembrolizumab, and atezolizumab) are the preferred choice for subsequent treatment of all histological subtypes because they have a higher survival rate, Longer response duration and less adverse events (AE) chemotherapy compared to cytotoxicity [6]. Pembrolizumab has been approved by the Food and Drug Administration (FDA) as subsequent therapy for patients with metastatic NSCLC whose disease has progressed after platinum-based chemotherapy if their tumors express PD-L1 [19].

Since the high incidence of lung cancer and high treatment costs have a significant impact on drug availability and the continued operation of the reimbursement fund, it is important to study the cost budget for lung cancer drugs. Budget impact analysis (BIA) is designed to measure the combined impact of the inclusion of a new pharmaceutical product on health care spending. Its main aim should be to complete cost-effectiveness analyses (CEAs) for reimbursement and coverage particularly for short- and mid-term budget planning. The structure of BIA is can be adjusted according to different needs for different countries as well as for time horizons, perspective and underlying diseases [19].

Many authorities have built up the BIA method by different criteria, but no one has provided a precise definition. Until 2007, the ISPOR Task Force presented guidance on methodologies for those reviewing the results of such analyses [20]. Then, the ISPOR Task Force developed good practice guidelines to improve high-quality BIAs [21]. At the same time, many countries and regions presented specific guidelines [22–24]. These guidelines reports the analytical framework, key elements and reporting format for BIAs, including research perspective, budget time horizon, drugs and other cost, new interventions, uncertainty analysis and validation, etc.

However, as far as we know, there has been no review examined budget impact analysis studies in the field of lung cancer. Many developed countries use evidence-based health technology assessment (HTA) methods to conduct cost–benefit analysis of clinically selected medical technologies as one of the main content of drug reimbursement recommendations [25–27]. In recent years, expensive anti-tumor drugs have been included in the catalogue of basic medical insurance drugs of China [28, 29], and the related drug price negotiations have made significant progress, which has a greater impact on the accessibility of drugs to patients and the continued operation of the medical insurance fund. Therefore, we focus on the budget impact analysis of anti-tumor drugs used to treat lung cancer worldwide, aiming to summarize key elements, results, and assess the extent to which international BIA guidelines are followed in these studies.

## Methods

We conducted a literature search of the databases Pubmed, EMBASE and the Cochrance Library to select articles on budget impact analysis for antitumor Drugs of lung cancer published in English from 1 January 2010 until 31 October 2019. Similarly, we used keywords to search on CNKI and Wanfang Data Knowledge Service Platform of China. The following search strategy was used: (Budget impact* OR budget impact model OR budget impact analysis OR pharmacoeconomics*) AND (Antineoplastic Agents [MeSH Major Topic]).We included studies reporting budget impact models or budget impact methods of anti-tumor drugs based on randomized controlled trials, cross-sectional studies, cohort studies, model studies, etc. And excluded studies that only examined efficacy, toxicity, studies that conducted only cost-effectiveness analyses, reviews, comments, meeting abstracts and BIAs of other cancer patients. Two independent reviewers performed title and abstract screening and full-text selection. A third author resolved the disagreement.

Based on the ISPOR Task Force guidelines [5], we developed evidence tables presenting a summary of how each study addressed the key elements, such as population size and characteristics, budget holder’s perspective, budget time horizon, model structure, clinical and cost data, cost calculation, uncertainty analysis, etc. And then we systematically extracted data and summarized our findings from all included studies in evidence tables.

Meanwhile, the level of adherence to the ISPOR Task Force guidelines [5] was summarized for the following items: Budget holder’s perspective; target population estimate; 1–5 years of budget time horizon; hypothetical scenario; control group; analysis framework description; data collection and sources; model verification and sensitivity analysis. This review was conducted according to the PRISMA (Preferred Reporting Items for Systematic Reviews and Meta-Analyses) guidelines [30]. The final included studies are all complete published literature, which may lead to inherent problems with publication bias.

## Results

**Fig. 1:**
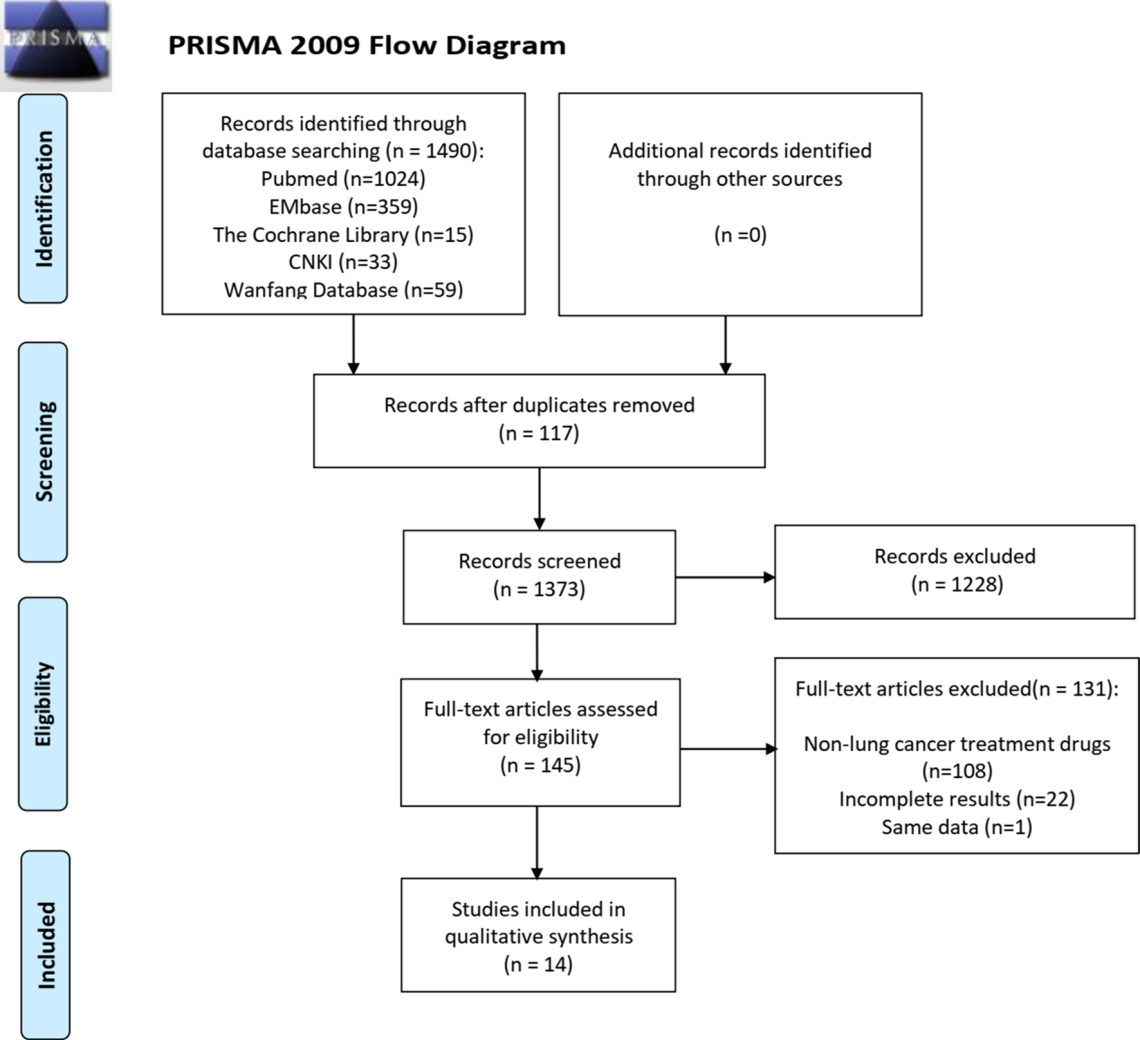
The flow chart of literature search strategy

Figure 1 summarizes the search strategy and its results. 1490 articles were initially retrieved through the identified keywords on the budget impact analysis of antitumor drugs. After deduplication (n = 117), screening the title and abstract (n = 1228) and the full text of remaining studies (n = 131), 14 items were finally included BIA studies of antitumor drugs for lung cancer. Half of 14 studies were from Europe and America, among which six studies were conducted for the US population [31–36] and one for Norway [28]. In the other half, there are 3 studies from Thailand [21, 22, 26] and 3 studies from China [25, 37, 38] and 1 study from South American countries [29].

Table 1 summarizes general information for the 14 selected BIA studies [31–36, 39–46]. Thirteen articles were studied based on models [31–36, 39–43, 45, 46], and one article was studied based on population randomized controlled trials [44]. The eligible populations were mainly chosen according to the coverage of the payer’s plan [31–36, 39–46]. There were thirteen studies restricted the target population based on the type and degree of lung cancer [31–36, 40–46]. Half of the 14 articles were from developed countries, of which 6 were from the US [31–36], 1 was from Norway [43]; the remaining 3 were from Thailand [39, 40, 42], 3 were from China [41, 45, 46], and 1 was from developing countries in South America [44].

**Table 1:** General information on selected BIAs

First author	Year	Country	Drugs	Research foundation	Population size, characteristics
Carlson [31]	2011	US	Erlotinib	Model	500,000 member US health plan; stage IIIB/IV NSCLC.
Farsai [39]	2011	Thailand	Pemetrexed	Model	Lung cancer patients in the hospital
Sumitra [40]	2012	Thailand	Gefitinib	Model	100,000 advanced NSCLC patients
Preeti [32]	2014	US	Erlotinib	Model	500,000 member health plan; with advanced NSCLC
Lisa [33]	2016	US	Ramucirumab + docetaxel	Model	150,000 patients in hospital; NSCLC Patients Receiving 2nd line Therapy
Mengyuan [41]	2016	China	Gefitinib	Model	74000 advanced NSCLC patients
Sumitra [42]	2017	Thailand	Crizotinib	Model	5183 NSCLC in lung cancer patient; aged ≧19 years
Daniel [34]	2017	US	Pembrolizumab	Model	19601 NSCLC patients with PD-L1 expression ≧ 50%
Jan [43]	2017	Norway	Pembrolizumab	Model	3035 cases; Patients with advanced NSCLC 2nd line treatment
Pedro [44]	2018	South American countries	Pembrolizumab	RCT	3043 participants; patients with NSCLC for immunotherapy
Christopher [35]	2018	US	Necitumumab	Model	100,000 plan participants; msqNSCLC patients receiving first-line chemotherapy; aged≧65 years
Jonathan [36]	2018	US	Afatinib	Model	Health plan for 1,000,000 people; metastatic NSCLC whose tumors have EGFR del19 or L858R mutations initiating first-line treatment; age ≥ 18 years
Xueyan [45]	2018	China	Icotinib	Model	73,400 patients with advanced NSCLC who were genetically tested and eligible for icotinib in the insured population
Jie [46]	2019	China	Afatinib	Model	45,554 patients with advanced NSCLC who were positive for EGFR mutation after chemotherapy in the insured population

*RCT* Randomized controlled trial, *NSCLC* non-small cell lung cancer, *msqNSCLC* metastatic squamous non-small cell lung cancer

Table 2 summarizes the budget impact analysis method and research results of the reviewed studies. We refined the key elements such as application model, research perspective, budget time frame, treatment plan, direct cost, indirect cost, clinical and cost data sources, and sensitivity analysis methods. In 14 studies, 13 studies were conducted from the payer and the hospital, only one study was conducted from the perspective of private insurance companies [32]. Four studies considered a hypothetical population from the perspective of health commercial planning [31, 32, 35, 36], which were conducted for US populations. There were 13 studies that determined the research model, most of which used the BIA cost calculation model (also called cost decision model) [31–33, 35, 36, 39, 41, 42, 45, 46], and only two of which used the Markov model [40] and Monte Carlo model [34] to simulate the disease process.

**Table 2:** Methodology and budget results of BIAs

First author	Model structure	Perspective	Budget time horizon	Treatment strategy	Cost calculation	Data sources	Sensitivity analysis	Research result
Carlson [31]	Cost calculator	Health plan	1 year	Erlotinib maintenance VS Erlotinib maintenance available	Direct cost + condition-related cost	RCT; Medicare and Medicaid and Drug price database; published literature	One-way	Erlotinib PMPM increase
Farsai [39]	Cost calculator	Hospital	4 years	Docetaxel VS Pemetrexed	Direct cost	Hospital inpatient database	One-way	Annual budget of Pemetrexed in hospital increase
Sumitra [40]	Markov model	CSMBS	5 years	Gefitinib;Erlotinib;Pemetrexed;Docetaxel	Direct cost + condition-related cost	RCT;NICE Public data	One-way	Cost saving of Gefitinib
Preeti [32]	Cost calculator	Private insurance company	1 year	Erlotinib;Pac + Car;Bev + Pac + Car;Pem + Car;Bev + Pem +Car	Direct cost	Hospital medical records; Medicare database	One-way	Erlotinib PMPM increase
Lisa [33]	Cost calculator	Hospital	1 year	Ram + Doc; Bev + Erl; Docetaxel; Erlotinib; Pemetrexed	Direct cost	Public Data;Electronic medical record data	NR	After ramucirumab adding to hospital formulary annual margin increase
Mengyuan [41]	Cost calculator	Medical insurance	5 years	Gefitinib VS chemotherapy	Direct cost	Tumor registration information; Project survey; Drug Market Trend Report	One-way	Budget savings after gefitinib is included in the medical insurance catalog
Sumitra [42]	Cost calculator	Payer	3 years	Crizotinib VS no crizotinib	Direct cost + condition-related cost	Thailand official database; Hospital database	One-way	Crizotinib PMPM increase
Daniel [34]	Monte Carlo model	Payer	1 year	Different dose control of the same drug	Direct cost	RCT/SEER and Medicare data	One-way	Budget savings for pembrolizumab of personalized dosing
Jan [43]	Cost minimization model	Payer	1 year	Pembrolizumab VS Docetaxel or pemetrexed	Direct cost + condition-related cost	RCT; National official data	One-way	Pembrolizumab 2nd-line treatment cost savings
Pedro [44]	NR	Payer	1 year	Cost-sharing, risk-sharing, payment-by-results, discount	Direct cost	RCT; National official data	NR	Budget impact of pembrolizumab in the first-line decreased through risk-sharing
Christopher [35]	Cost calculator	U.S. commercial and Medicare health plans	3 years	Before and after adoption of Neci + GCis	Direct cost + condition-related cost	RCT; U.S. prescription information and clinical guidelines; Public data	One-way	Neci + GCis annual budget increase
Jonathan [36]	Decision model	U.S. commercial health plan	5 years	Constant Uptake VS Increase in Afatinib Uptake	Direct cost + condition-related cost	Public data; Project survey; Published literature	One-way	Afatinib annual budget increase
Xueyan [45]	Cost calculator	Medical insurance	5 years	Icotinib VS chemotherapy	Direct cost	Published literature; Project survey data	One-way	Icotinib annual medical insurance cost budget savings
Jie [46]	Cost calculator	Medical insurance	3 years	With and without afatinib in the Chinese national reimbursement system	Direct cost	Published literature; Clinical expert opinion	One-way	Afatinib annual medical insurance budget expenditure decreases

*CSMBS* Civil Servant Medical Benefit Scheme, *Pac* paclitaxel, *Car* carboplatin, *Bev* bevacizumab, *Pem* pemetrexed, *Ram* ramucirumab, *Doc* Docetaxel, *Neci* necitumumab, *GCis* gemcitabine and cisplatin, *NR* no report

The budget time horizon determined for the model is based on the requirements of the budget holder. The budget time horizon of the included studies was concentrated in 1–5 years, and there were 6 studies with shorter budget periods, only 1 year [31–34, 43, 44]. Most studies presented a budget time horizon of 3 years or more, 3 studies presented a budget time horizon of 3 years [35, 42, 46], 1 study presented a budget time horizon of 4 years [39]. Four studies presented a five-year budget time horizon [36, 40, 41, 45].

Treatment strategy in the reviewed studies were stated by 14 studies. Most studies compared research drug between A treatment strategy and B treatment strategy under different scenarios [31–33, 35, 39–43, 45, 46]. Two studies compared different doses of the same drug setting in the base case analysis [34, 36]. One study did not compare treatment strategies, but divided strategies according to different payment methods, including cost-sharing, risk-sharing, payment-by-results and discount [44].

Cost calculation in the reviewed studies was summarized as direct cost and condition-related cost. Direct cost included in the selection of cost accounting mainly take into account the cost of drugs and the cost of genetic testing. Adverse event cost and management costs are called condition-related cost. Eight studies only calculated direct costs [32–34, 39, 41, 44–46], and six studies chose direct cost plus condition-related cost in cost calculation [31, 35, 36, 40, 42, 43]; The ISPOR Task Force guidelines [5] recommend that data should come from the best available sources and should be thoroughly quoted to support transparency and reproducibility. The 14 studies included in the study all clearly stated the source of clinical and cost data [31–36, 39–46], typically from surveys, official national data, published literature, and a few from randomized controlled trials.

Of the 14 reviewed studies, twelve studies were subjected to sensitivity analysis, and all methods were conducted using one-way sensitivity analysis [31, 32, 34–36, 39–43, 45, 46]. However, the selection of sensitivity analysis parameters is inconsistent, such as whether to analyze the treatment cycle. Regarding research on the budget impact analysis of drugs, the ISPOR Task Force guidelines did not recommend discounting [5], so we did not take into account discounting.

As for the result indicators, we found that the included studies were all presented in the form of budget amounts. Six studies indicated budget results increased [31, 32, 35, 36, 39, 42]. One of the studies explained that it was mainly due to the increase in the cost of drugs, that was, the increase in the cost of progression-free survival and treatment cycle extension [42]. The other study showed that the higher incremental budget in medical insurance was due to the higher incidence of metastatic squamous non-small cell lung cancer among elderly medical insurance patients [35]. On the contrary, six studies budget decreased [40, 41, 43–46]. One of the studies used the expected annual margin between costs and reimbursement to explain the results of budget impact analysis [33]. One study in the United States indicated that personalized-dosing of drug resulted in cost savings over fixed-dosing [34].

Table 3 provides a summary of the quality evaluation according to the ISPOR Task Force guidelines [5]. The consistency of the included BIAs and ISPOR Task Force guidelines indicates that the overall quality of the included studies was good, and 9 of included studies followed at least 8 of the guidelines (≥ 88.9%) [32–36, 40, 41, 43, 45], 4 studies [31, 39, 42, 46] followed 7 items (77.8%) in the guidelines. Only 1 study [44] followed less than 5 items (44.4%). Overall, most studies did not report model validation, and only 14.3% of the studies conducted model validation.

**Table 3:** Quality evaluation of BIAs

First author	Perspective	Target population estimate	1–5 years of budget time horizon	Hypothetical scenario	Comparator	Frame description	Data collection and sources	Validation	Sensitivity analysis
Carlson [31]	√	√	√	√	√		√		√
Farsai [39]	√	√	√	√	√		√		√
Sumitra [40]	√	√	√	√	√	√	√		√
Preeti [32]	√	√	√	√	√	√	√		√
Lisa [33]	√	√	√	√	√	√	√	√	
Mengyuan [41]	√	√	√	√	√	√	√		√
Sumitra [42]	√	√	√	√	√		√		√
Daniel [34]	√	√	√	√	√	√	√		√
Jan [43]	√	√	√	√	√	√	√	√	√
Pedro [44]	√	√	√				√		
Christopher [35]	√	√	√	√	√	√	√		√
Jonathan [36]	√	√	√	√	√	√	√		√
Xueyan [45]	√	√	√	√	√	√	√		√
Jie [46]	√	√	√	√	√		√		√

## Discussion

We systematically reviewed the budget impact analysis for anti-tumor drugs of lung cancer, which from Europe, America, Asia and South America, covering a wide range of areas. And summarized methodological elements and research results to provide reference for the budget impact analysis of high-value drugs. Firstly, we found from these reviews of the design of published budget-impact models was that, despite published guidelines for budget-impact analysis, there were still significant differences in the included studies. Many countries and regions had issued budget impact analysis guidelines, such as Canada [6], France [7], and Ireland [8]. The latest China Guidelines for Pharmacoeconomic Evaluations was published in 2019, which included budget impact analysis methods and rules [47]. The BIA method has not been specified in a unified and standardized form, so there were significant differences between BIA studies.

Vooren [48] considered that BIA was not a mature technology in the literature in 2013, and many published studies have not yet reached acceptable quality. In particular, the short-term budget savings of high-value drugs might be caused by the bias of pharmaceutical companies. Mauskopf [37] found that many budget impact analyses’ designs were still different even for those analyses performed for a new drug for the same type of disease. Beate Jahn [38] also considered that best-practice guidelines were necessary to ensure high-quality analyses. Although we agreed on the importance of a mature framework for BIA, it was more important to implement the operations of BIA. Such as pembrolizumab, two studies in Norway [43] and south America [44] showed that pembrolizumab’s budget decreased, while Thailand’s [39] BIA study recognized that budget of pembrolizumab increased significantly. Erlotinib’s budget impact analysis results were consistent. Coincidentally, both of these two BIAs of erlotinib were from the United States. The consistent result may be related not only to the uniform BIA guidelines in the U.S., but also to the drug reimbursement policy in the U.S.. We also observed the results of BIAs showing a continuous decline in the health insurance budget for different drugs in China consistently [41, 45, 46]. We thought it was not only related to China’s special medical insurance drug policy, but also related to the input of related parameters of the BIA model. Therefore, we suggested that BIA research should carefully consider the size of the population based on real-world data, rather than model simulation, to make the budget impact results more realistic in order to provide a realistic reference for decision-making by decision-making departments.

From the Thai payer perspective, gefitinib was a dominant cost saving strategy compared with docetaxel for the second-line treatment of advanced NSCLC [40]. Since gefitinib had been reapproved through the FDA’s Phase IV study, it was now more accepted and very well tolerated by most patients [6]. New drugs such as gefitinib should be paid more attention to comparing budget impact analysis of different drugs to ensure that there was no financial burden on the use of new drugs. Our review suggested that reasonable comparators should be set up based on the clinical pathway, and this kind of research had greater clinical reference value.

Meanwhile, the short-term increase or decrease in the budget impact results should not directly determine the inclusion or exclusion of drugs in the medical insurance catalog. On the contrary, the long-term budget impact of drugs should be considered. For instance, the impact of new drugs with good effects on the short-term budget has increased, but in the long run, they will save costs, can still be considered within the control of the medical insurance fund, and vice versa. Although the results of budget impact analysis of icotinib showed a downward trend, the extent of decline had decreased year by year [45]. Through the long-term budget time horizon research, the trend of budget increase or decrease could be seen to predict whether the fund was affordable.

We found that most of the included studies did not undergo model validation. Only two studies stated that the validity of the BIA model was discussed with clinical experts and relevant researchers [33, 43]. ISPOR had already put forward requirements for the validity verification of the BIA model in 2014 [5], and the latest economic evaluation guidelines in China had also made requirements for the validation of the BIA model, including face validation, technical validation and external validation [47]. Obviously, model validation should be a key element to ensure the accuracy of BIA research.

Then, we identified the following key elements for the design of the budget impact model: the BIA budget time horizon should be considered for at least the next 3-5 years; Considering the differences in previous BIAs, our review suggested that reasonable comparators should be set up based on real-world policy and the clinical pathway; Sensitivity analysis could consider the use of multi-factor analysis, which was sufficient to consider the correlation between various factors; Model validation and sensitivity analysis should be carried out to ensure the effectiveness of BIA research [49]; And the key elements should cooperate with each other to ensure effective budget impact analysis.

Compared with previous BIA reviews [37, 38, 48], this systematic review has been focuses on lung cancer drugs specifically. We have summarized the key elements to ensure the quality of BIA research comprehensively. In addition, we concluded the budget results of the included studies to provide a comprehensive reference for BIA studies of high-value drugs of lung cancer. Our systematic review has several limitations. First, some studies may have been missed because they were indexed in other databases or were published by conference did not appear in the journal publications retrieved by this study. Second, due to the language limitation, our systematic review included studies published in English and Chinese. References were retrieved from three international databases and two Chinese databases (Pubmed, EMBASE, the Cochrance Library, CNKI and Wanfang Data Knowledge Service Platform of China). Third, the search time was limited to 2010-2019. Due to the variety of anti-tumor drug studies, the search time could not be exhausted, so we chose to search the relevant studies in the past decade. Fourth, in the summary of the results, the results were evaluated only from the vertical perspective of increase and decrease, and the budget results were not uniformly converted into international currency forms such as US dollars [50], so that there was no lateral difference evaluation of the extrapolation of results.

## Conclusion

Although most of the included BIA studies are conducted from the perspective of payers, they have different methodological framework for recommended chemotherapy, targeted therapy, and immunotherapy agents for the treatment of lung cancer. For the same drugs, the results of budgetary effects are not consistent in different country. The budget impact analysis of high-value drugs such as anti-tumor drugs should be conducted more objectively, and the accuracy of parameters needs to be more strictly guaranteed. The high-quality BIAs should be based on real-world data to provide reliable results for decision-making departments. Furthermore, it is more worthy of attention that the budgetary impact of the same drug is not always consistent over time, so access to drugs should be measured in the long run.

## Supplementary Information

**Additional file 1: Table S1.** PRISMA 2009 checklist.

## Data Availability

All data involved in this study are included in this published article and its supplementary information files.

## Abbreviations

BIAs: Budget impact analyses
CEAs: Cost-effectiveness analyses
NSCLC: Non-small cell lung cancer
SCLC: Small cell lung cancer
EGFR: Epidermal Growth Factor Receptor
TKIs: Tyrosine kinase inhibitors
ALK: Anaplastic lymphoma kinase
CNKI: China national knowledge infrastructure

## References

[1] Bray F, Ferlay J, Soerjomataram I, Siegel RL, Torre LA, Jemal A (2018). Global cancer statistics 2018: GLOBOCAN estimates of incidence and mortality worldwide for 36 cancers in 185 countries. CA Cancer J Clin.

[2] Luengo-Fernandez R, Leal J, Gray A, Sullivan R (2013). Economic burden of cancer across the European Union: a population-based cost analysis. Lancet Oncol..

[3] Travis WD, Brambilla E, Nicholson AG (2015). The 2015 World Health Organization Classification of Lung Tumors: impact of genetic, clinical and radiologic advances since the 2004 classification. J Thorac Oncol.

[4] Travis WD, Brambilla E, Burke AP (2015). WHO classification of tumours of the lung, pleura, thymus and heart.

[5] Howlader N, Noone AM, Krapcho M, et al. SEER Cancer Statistics Review,1975-2013, based on November 2015 SEER data submission, posted to the SEER web site, April 2016. Bethesda, MD: National Cancer Institute; 2016. Available at: http://seer.cancer.gov/csr/1975_2013/. Accessed 3 March 2017.

[6] Ettinger DS, Wood DE, Aisner DL (2017). Non-small cell lung cancer, Version 5.2017, NCCN Clinical Practice Guidelines in Oncology. J Natl Compr Canc Netw.

[7] Langer CJ (2013). Epidermal growth factor receptor inhibition in mutation-positive non-small-cell lung cancer: is afatinib better or simply newer?. J Clin Oncol.

[8] Kwak EL, Bang YJ, Camidge DR (2010). Anaplastic lymphoma kinase inhibition in non-small-cell lung cancer. N Engl J Med.

[9] Shaw AT, Yeap BY, Mino-Kenudson M (2009). Clinical features and outcome of patients with non-small-cell lung cancer who harbor EML4-ALK. J Clin Oncol.

[10] Burotto M, Manasanch EE, Wilkerson J, Fojo T (2015). Gefitinib and erlotinib in metastatic non-small cell lung cancer: a meta-analysis of toxicity and efficacy of randomized clinical trials. Oncologist.

[11] Haspinger ER, Agustoni F, Torri V (2015). Is there evidence for different effects among EGFR-TKIs? Systematic review and meta-analysis of EGFR tyrosine kinase inhibitors (TKIs) versus chemotherapy as first-line treatment for patients harboring EGFR mutations. Crit Rev Oncol Hematol.

[12] Cohen MH, Johnson JR, Chen YF (2005). FDA drug approval summary: erlotinib (Tarceva) tablets. Oncologist.

[13] Eberhard DA, Johnson BE, Amler LC (2005). Mutations in the epidermal growth factor receptor and in KRAS are predictive and prognostic indicators in patients with non-small-cell lung cancer treated with chemotherapy alone and in combination with erlotinib. J Clin Oncol.

[14] Sequist LV, Joshi VA, Janne PA (2007). Response to treatment and survival of patients with non-small cell lung cancer undergoing somatic EGFR mutation testing. Oncologist.

[15] Inoue A, Kobayashi K, Usui K (2009). First-line gefitinib for patients with advanced non-small-cell lung cancer harboring epidermal growth factor receptor mutations without indication for chemotherapy. J Clin Oncol.

[16] Nelson V, Ziehr J, Agulnik M, Johnson M (2013). Afatinib: emerging next-generation tyrosine kinase inhibitor for NSCLC. Onco Targets Ther..

[17] De Greve J, Teugels E, Geers C (2012). Clinical activity of afatinib (BIBW 2992) in patients with lung adenocarcinoma with mutations in the kinase domain of HER2/neu. Lung Cancer.

[18] Khozin S, Blumenthal GM, Jiang X (2014). U.S. Food and Drug Administration approval summary: erlotinib for the first-line treatment of metastatic non-small cell lung cancer with epidermal growth factor receptor exon 19 deletions or exon 21 (L858R) substitution mutations. Oncologist.

[19] Sul J, Blumenthal GM, Jiang X (2016). FDA approval summary:pembrolizumab for the treatment of patients with metastatic non-small cell lung cancer whose tumors express programmed death-ligand 1. Oncologist.

[20] Trueman P, Drummond M, Hutton J (2001). Developing guidance for budget impact analysis. Pharmacoeconomics..

[21] Mauskopf JA, Sullivan SD, Annemans L, Caro J, Mullins CD, Nuijten M, Orlewska E, Watkins J, Trueman P (2007). Principles of good practice for budget impact analysis: report of the ISPOR Task Force on good research practices—budget impact analysis. Value Health..

[22] Sullivan SD, Mauskopf JA, Augustovski F (2014). Budget impact analysis—principles of good practice: report of the ISPOR 2012 Budget Impact Analysis Good Practice II Task Force. Value Health.

[23] Marshall DA, Douglas PR, Drummond MF (2008). Guidelines for conducting pharmaceutical budget impact analyses for submission to public drug plans in Canada. Pharmacoeconomics.

[24] Ghabri S (2018). Budget impact analysis guidelines developed in France[J]. PharmacoEconomics & Outcomes News.

[25] Quality Authority (HIQA). Guidelines for the budget impact analysis of health technologies in Ireland 2010[J]. health information & quality authority, 2010.

[26] Australian Government Department of Health. The Pharmaceutical benefits scheme: an overview[EB/OL].[2016-04- 20].http://webarchive.nla.gov.au/gov/20020919043036/; http://www.aph.gov.au:80/Library/intguide/SP/pbs.htm. Accessed 20 Apr 2016.

[27] Drummond MF, Mason AR (2007). European perspective on the costs and cost-effectiveness of cancer therapies. J Clin Oncol.

[28] Sullivan SD, Watkins J, Sweet B, Ramsey SD (2009). Health technology assessment in health-care decisions in the United States. Value Health.

[29] Notice of the Ministry of Human Resources and Social Security on the inclusion of 36 drugs in the category B of the National Basic Medical Insurance, Industrial Injury Insurance and Maternity Insurance Drug List [EB/OL]. http://www.mohrss.gov.cn/gkml/zlbmxgwj/ylbx_3063/201707/t20170718_274153.html.

[30] Moher D, Liberati A, Tetzlaff J, Altman DG (2009). Preferred reporting items for systematic reviews and meta-analyses: the PRISMA statement. PLoS Med..

[31] Carlson JJ, Wong WB, Veenstra DL (2011). Budget impact of erlotinib for maintenance therapy in advanced non-small cell lung cancer. J Med Econ.

[32] Bajaj PS, Veenstra DL, Goertz HP (2014). Targeted erlotinib for first-line treatment of advanced non-small cell lung cancer: a budget impact analysis. J Med Econ.

[33] Hess LM, Cinfio FN, Wetmore S (2016). Enhancing the budget impact model for institutional use: a tool with practical applications for the hospital oncology pharmacy. Hosp Pharm.

[34] Goldstein DA, Gordon N, Davidescu M, et al. A Phamacoeconomic analysis of personalized dosing vs fixed dosing of pembrolizumab in firstline PD-L1-positive non-small cell lung cancer. J Natl Cancer Inst, 2017, 109(11). 10.1093/jnci/djx063 29059432

[35] Bly CA, Molife C, Brown J (2018). The budget impact of including necitumumab on the formulary for first-line treatment of metastatic squamous non-small cell lung cancer: U.S. commercial payer and medicare perspectives. J Manag Care Spec Pharm.

[36] Graham J, Earnshaw S, Burslem K (2018). Budget impact analysis of afatinib for first-line treatment of patients with metastatic non-small cell lung cancer with epidermal growth factor receptor exon 19 deletions or exon 21 substitution mutations in a U.S. health plan. J Manag Care Spec Pharm.

[37] Mauskopf J, Earnshaw S (2016). A methodological review of US budget-impact models for new drugs. Pharmacoeconomics.

[38] Jahn B, Todorovic J, Bundo M (2019). Budget impact analysis of cancer screening: a methodological review. Appl Health Econ Health Policy..

[39] Chanjaruporn F, Roughead EE, Sooksriwong C (2011). Budget impact analysis of pemetrexed introduction: case study from a teaching hospital perspective, Thailand. J Med Assoc Thai.

[40] Thongprasert S, Tinmanee S, Permsuwan U (2012). Cost-utility and budget impact analyses of gefitinib in second-line treatment for advanced non-small cell lung cancer from Thai payer perspective. Asia Pac J Clin Oncol.

[41] Mengyuan Tian, Xiao Yin, Yuxiao Zhang, Fang Xin Hu, Jianglin Cui Dan, Zongfu Mao (2016). Analysis of the effect of gefitinib on the medical insurance budget of advanced non-small cell lung cancer. Health Econ Res.

[42] Thongprasert S, Permsuwan U (2017). Crizotinib treatment for advanced non-small-cell lung cancer patients: a budget impact analysis based in Thailand. Curr Med Res Opin.

[43] Norum J, Antonsen MA, Tollali T (2017). Pembrolizumab as second-line therapy in non-small cell lung cancer in northern Norway: budget impact and expected gain-a model-based analysis. ESMO Open.

[44] Aguiar P, Giglio AD, Perry LA (2018). Cost-effectiveness and budget impact of lung cancer immunotherapy in South America: strategies to improve access[J]. Immunotherapy.

[45] Xueyan Luo, Quan Yuan, Wenbing Yao (2018). Analysis of the budget impact of icotinib for the treatment of advanced non-small cell lung cancer. Chin New Drugs J.

[46] Jie F, Bin W (2019). Analysis of the budget effect of afatinib in the treatment of patients with advanced non-small cell lung cancer with EGFR mutation. China Modern Applied Pharmacy.

[47] Liu G (2019). China guidelines for pharmacoeconomic evaluations.

[48] Vooren KVD, Duranti S (2014). A critical systematic review of budget impact analyses on drugs in the EU countries. Appl Health Econ Health Policy.

[49] Eddy DM, Hollingworth W, Caro JJ (2012). ISPOR-SMDM modeling good Research Practice Task Force. Model transparency and validation: a report of the ISPOR-SMDM Modeling Good Research Practices Task Force—7. Value Health.

[50] Grochtdreis T, König HH, Dobruschkin A, vonAmsberg G, Dams J (2018). Cost-effectiveness analyses and cost analyses in castration-resistant prostate cancer: a systematic review. PLoS ONE.

[51] Notice of the National Medical Security Administration on including 17 anticancer drugs into the category B of the national basic medical insurance, industrial injury insurance and maternity insurance drug catalog [EB/OL]. http://www.nhsa.gov.cn/art/2018/10/10/art_19_397.html.

